# COVID-19 associated pulmonary aspergillosis in critically-ill patients: a prospective multicenter study in the era of Delta and Omicron variants

**DOI:** 10.1186/s13613-024-01296-0

**Published:** 2024-04-24

**Authors:** Pierre Bay, Etienne Audureau, Sébastien Préau, Raphaël Favory, Aurélie Guigon, Nicholas Heming, Elyanne Gault, Tài Pham, Amal Chaghouri, Matthieu Turpin, Laurence Morand-Joubert, Sébastien Jochmans, Aurélia Pitsch, Sylvie Meireles, Damien Contou, Amandine Henry, Adrien Joseph, Marie-Laure Chaix, Fabrice Uhel, Damien Roux, Diane Descamps, Malo Emery, Claudio Garcia-Sanchez, David Levy, Sonia Burrel, Julien Mayaux, Antoine Kimmoun, Cédric Hartard, Frédéric Pène, Flore Rozenberg, Stéphane Gaudry, Ségolène Brichler, Antoine Guillon, Lynda Handala, Fabienne Tamion, Alice Moisan, Thomas Daix, Sébastien Hantz, Flora Delamaire, Vincent Thibault, Bertrand Souweine, Cecile Henquell, Lucile Picard, Françoise Botterel, Christophe Rodriguez, Armand Mekontso Dessap, Jean-Michel Pawlotsky, Slim Fourati, Nicolas de Prost

**Affiliations:** 1 Médecine Intensive Réanimation, Hôpitaux Universitaires Henri Mondor, Assistance Publique-Hôpitaux de Paris (AP-HP), CHU Henri Mondor, 51, Av. de Lattre de Tassigny, CEDEX, 94010 Créteil, France; 2 Groupe de Recherche Clinique CARMAS, Université Paris-Est-Créteil (UPEC), Créteil, France; 3 Université Paris-Est-Créteil (UPEC), Créteil, France; 4 IMRB INSERM U955, Team “Viruses, Hepatology, Cancer”, Créteil, France; 5 IMRB INSERM U955, Team CEpiA, Créteil, France; 6 Unité de Recherche Clinique, Department of Public Health, Hôpitaux Universitaires Henri Mondor, Assistance Publique-Hôpitaux de Paris (AP-HP), Créteil, France; 7 U1167-RID-AGE Facteurs de Risque et Déterminants Moléculaires des Maladies Liées au Vieillissement, University Lille, Inserm, CHU Lille, Institut Pasteur de Lille, 59000 Lille, France; 8 Service de Virologie, CHU de Lille, 59000 Lille, France; 9 Médecine Intensive Réanimation, Hôpital Raymond Poincaré, Assistance Publique-Hôpitaux de Paris (AP-HP), Garches, France; 10 Laboratoire de Virologie, Hôpital Ambroise Paré, Assistance Publique-Hôpitaux de Paris (AP-HP), Boulogne, France; 11 Service de Médecine Intensive-Réanimation, Assistance Publique-Hôpitaux de Paris, Hôpital de Bicêtre, DMU 4 CORREVE Maladies du Cœur et des Vaisseaux, FHU Sepsis, Le Kremlin-Bicêtre, France; 12 Inserm U1018, Equipe d’Epidémiologie Respiratoire Intégrative, CESP, 94807 Villejuif, France; 13 Laboratoire de Virologie, Hôpital Paul Brousse, Assistance Publique-Hôpitaux de Paris, Villejuif, France; 14 Centre de Recherche Saint-Antoine INSERM, Médecine Intensive Réanimation, Hôpital Tenon, Assistance Publique-Hôpitaux de Paris, Sorbonne Université, Paris, France; 15 INSERM, Institut Pierre Louis d’Epidémiologie et de Santé Publique, Sorbonne Université, Paris, France; 16 Laboratoire de Virologie, Hôpital Saint-Antoine, Assistance Publique-Hôpitaux de Paris, 75012 Paris, France; 17 Service de Réanimation Polyvalente, Hôpital Marc Jacquet, Melun, France; 18 Laboratoire de Microbiologie, Hôpital Marc Jacquet, Melun, France; 19 Service de Réanimation Médico-Chirurgicale, Assistance Publique-Hôpitaux de Paris, Hôpital Ambroise Paré, Boulogne, France; 20 Service de Réanimation, Hôpital Victor Dupouy, Argenteuil, France; 21 Service de Virologie, Hôpital Victor Dupouy, Argenteuil, France; 22 Médecine Intensive Réanimation, Hôpital Saint-Louis, Assistance Publique-Hôpitaux de Paris, Paris, France; 23 Inserm HIPI, Université Paris Cité, 75010 Paris, France; 24 Laboratoire de Virologie, Hôpital Saint-Louis, Assistance Publique-Hôpitaux de Paris, 75010 Paris, France; 25 DMU ESPRIT, Service de Médecine Intensive Réanimation, Université Paris Cité, APHP, Hôpital Louis Mourier, Colombes, France; 26 INSERM U1151, CNRS UMR 8253, Department of Immunology, Infectiology and Hematology, Institut Necker-Enfants Malades (INEM), Paris, France; 27 IAME INSERM UMR 1137, Service de Virologie, Hôpital Bichat-Claude Bernard, Assistance Publique-Hôpitaux de Paris, Université Paris Cité, Paris, France; 28 Service de Réanimation, Hôpital Saint-Camille, Bry-Sur-Marne, France; 29 Laboratoire de Biologie, Hôpital Saint-Camille, Bry-Sur-Marne, France; 30 Assistance Publique-Hôpitaux de Paris, Hôpital Pitié-Salpêtrière, Réanimation Médicale, Sorbonne Université, Paris, France; 31 Service de Virologie, CHU de Bordeaux et CNRS UMR 5234, Fundamental Microbiology and Pathogenicity, Université de Bordeaux, Bordeaux, France; 32 Département de Virologie, Hôpital Pitié-Salpêtrière, Assistance Publique-Hôpitaux de Paris (AP-HP), Paris, France; 33 Assistance Publique-Hôpitaux de Paris, Hôpital Pitié-Salpêtrière, Médecine Intensive Réanimation, Sorbonne Université, Paris, France; 34 CHRU de Nancy, Médecine Intensive et Réanimation Brabois, Université de Lorraine, Vandœuvre-Lès-Nancy, France; 35 INSERM U942 and U1116, F-CRIN-INIC RCT, Vandœuvre-Lès-Nancy, France; 36 Service de Virologie, CHRU de Nancy, Vandœuvre-Lès-Nancy, France; 37 Médecine Intensive Réanimation, Hôpital Cochin, Assistance Publique-Hôpitaux de Paris, Paris, France; 38 Laboratoire de Virologie, Hôpital Cochin, Assistance Publique-Hôpitaux de Paris, Paris, France; 39 Service de Réanimation, Hôpital Avicenne, Assistance Publique-Hôpitaux de Paris, Bobigny, France; 40 Laboratoire de Virologie, Hôpital Avicenne, Assistance Publique-Hôpitaux de Paris, Bobigny, France; 41 Intensive Care Unit, Tours University Hospital, Research Center for Respiratory Diseases (CEPR), INSERM U1100, University of Tours, Tours, France; 42 INSERM U1259, Université de Tours, Tours, France; 43 CHRU de Tours, National Reference Center for HIV-Associated Laboratory, Tours, France; 44 Service de Médecine Intensive-Réanimation, CHU De Rouen, Rouen, France; 45 INSERM, Normandie Univ, DYNAMICURE UMR 1311, CHU Rouen, Department of Virology, Univ Rouen Normandie, Université de Caen Normandie, 76000 Rouen, France; 46 Réanimation Polyvalente, INSERM CIC 1435 and UMR 1092, CHU Limoges, Limoges, France; 47 French National Reference Center for Herpesviruses, Bacteriology, Virology, Hygiene Department, CHU Limoges, 87000 Limoges, France; 48 INSERM, RESINFIT, U1092, 87000, Limoges, France; 49 CHU Rennes, Maladies Infectieuses et Réanimation Médicale, Rennes, France; 50 Laboratoire de Virologie, CHU Rennes, 35000 Rennes, France; 51 Inserm, EHESP, Irset (Institut de Recherche en Santé, Environnement et Travail) UMR_S 1085, Univ Rennes, 35000 Rennes, France; 52 Service de Médecine Intensive et Réanimation, CHU Clermont-Ferrand, Clermont-Ferrand, France; 53 3IHP, Service de Virologie, CHU Clermont-Ferrand, Clermont-Ferrand, France; 54 Département d’Anesthésie Réanimations Chirurgicales, Hôpitaux Universitaires Henri Mondor, Assistance Publique-Hôpitaux de Paris (AP-HP), Créteil, France; 55 Department of Virology, Hôpitaux Universitaires Henri Mondor, Assistance Publique-Hôpitaux de Paris, Créteil, France

**Keywords:** COVID-19, Invasive pulmonary aspergillosis, Intensive care unit, SARS-CoV-2, Omicron, COVID-19 associated pulmonary aspergillosis, Acute respiratory distress syndrome

## Abstract

**Background:**

During the first COVID-19 pandemic wave, COVID-19-associated pulmonary aspergillosis (CAPA) has been reported in up to 11–28% of critically ill COVID-19 patients and associated with increased mortality. As new SARS-CoV-2 variants emerged, the characteristics of critically ill COVID-19 patients have evolved, particularly in the era of Omicron. The purpose of this study is to investigate the characteristics of CAPA in the era of new variants.

**Methods:**

This is a prospective multicenter observational cohort study conducted in France in 36 participating intensive care units (ICU), between December 7th, 2021 and April 26th 2023. Diagnosis criteria of CAPA relied on European Confederation of Medical Mycology (ECMM)/International Society for Human & Animal Mycology (ISHAM) consensus criteria.

**Results:**

566 patients were included over the study period. The prevalence of CAPA was 5.1% [95% CI 3.4–7.3], and rose to 9.1% among patients who required invasive mechanical ventilation (IMV). Univariable analysis showed that CAPA patients were more frequently immunosuppressed and required more frequently IMV support, vasopressors and renal replacement therapy during ICU stay than non-CAPA patients. SAPS II score at ICU admission, immunosuppression, and a SARS-CoV-2 Delta variant were independently associated with CAPA in multivariable logistic regression analysis. Although CAPA was not significantly associated with day-28 mortality, patients with CAPA experienced a longer duration of mechanical ventilation and ICU stay.

**Conclusion:**

This study contributes valuable insights into the prevalence, characteristics, and outcomes of CAPA in the era of Delta and Omicron variants. We report a lower prevalence of CAPA (5.1%) among critically-ill COVID-19 patients than previously reported, mainly affecting intubated-patients. Duration of mechanical ventilation and ICU stay were significantly longer in CAPA patients.

**Supplementary Information:**

The online version contains supplementary material available at 10.1186/s13613-024-01296-0.

## Introduction

The societal and individual consequences of pneumonia caused by respiratory viruses, notably due to influenza virus and SARS-CoV-2, are well established. Patients with severe pneumonia may develop acute respiratory failure and require admission to the intensive care unit (ICU). Replication of a respiratory virus in the lower respiratory tract and severe inflammation associated with immune cell infiltration lead to gas exchanges impairment. Viral pneumonia increases patients’ susceptibility to bacterial and fungal superinfections, including invasive pulmonary aspergillosis [1, 2]. Influenza-associated pulmonary aspergillosis (IAPA) has been reported in up to 19–25% of critically ill patients with influenza, associated with poor outcomes [2, 3]. Coronavirus disease 2019 (COVID-19)-associated pulmonary aspergillosis (CAPA) has similarly emerged as an important coinfection in critically ill patients with COVID-19. The diagnostic criteria for these co-infections combine clinical, radiological, mycological, and histological criteria [4]. A recent autopsy study on patients infected with influenza and COVID-19 confirmed the invasive nature of the infection and the similarity of histological lesions observed in both IAPA and CAPA cases [5]. A multicenter French study conducted during the first wave revealed that 15% of critically ill patients with COVID-19 requiring invasive mechanical ventilation (IMV) fulfilled the diagnostic criteria for CAPA, which was also associated with poor outcomes [1]. In addition to the prognostic impact of CAPA, studies conducted during the first wave identified host-related risk factors of CAPA, including age, concomitant treatment with corticosteroids and tocilizumab, and prolonged duration of mechanical ventilation [1, 6].

As various SARS-CoV-2 variants have emerged along with the epidemic waves, the characteristics of critically ill COVID-19 patients significantly evolved, especially since the Omicron era: reduced use of IMV, higher rate of immunosuppressed patients, and different treatment approaches as compared to the first COVID-19 wave [7]. The purpose of this study is to investigate the characteristics of CAPA with the emergence of Delta variant followed by Omicron and related sublineages, identify potential predictive factors and assess its prognosis impact.

## Methods

### Patients and clinical data

This study is a prospective multicenter observational cohort study. Patients admitted between December 7th, 2021 and April 26th, 2023 in one of the 36 participating ICUs (including 19 from the Greater Paris area) were eligible for inclusion in the SEVARVIR cohort study (NCT05162508) if they presented the following inclusion criteria: age ≥ 18 years, SARS-CoV-2 infection confirmed by a positive reverse transcriptase-polymerase chain reaction (RT-PCR) in nasopharyngeal swab samples, admission in the ICU for acute respiratory failure (i.e., peripheral oxygen saturation (SpO2) ≤ 90% and need for supplemental oxygen or any kind of ventilator support), patient or next of kin informed of study inclusion. Patients with SARS-CoV-2 infection but no acute respiratory failure or with a RT-PCR cycle threshold (Ct) value > 32 in nasopharyngeal swabs were not included. Over the study period, 47% of participating ICUs had rooms with negative pressure, accounting for a total of 37% of the rooms. The study was approved by the Comité de Protection des Personnes Sud-Méditerranée I (N° EudraCT/ID-RCB: 2021-A02914-37). Informed consent was obtained from all patients or their relatives.

Demographics, clinical and laboratory variables were recorded upon ICU admission and during ICU stay. Patients’ frailty was assessed using the Clinical Frailty Scale [8]. The severity of the disease upon ICU admission was assessed using the World Health Organization (WHO) 10-point ordinal scale [9], the sequential organ failure assessment (SOFA) score [10], and the simplified acute physiology score (SAPS) II score [11]. Acute respiratory distress syndrome (ARDS) was defined according to the Berlin definition [12]. CAPA diagnosis work-up was at the initiative of the attending clinician in our study (i.e., targeted sampling strategy). The diagnosis and classification (i.e., proven, probable or possible) of CAPA relied on ECMM/ISHAM (European Confederation of Medical Mycology, the International Society for Human and Animal Mycology) international consensus criteria (Additional file 2: Table S1) [4].

### SARS-CoV-2 variant determination

Full-length SARS-CoV-2 genomes from all included patients were sequenced by means of next-generation sequencing. Briefly, viral RNA was extracted from nasopharyngeal swabs in viral transport medium using NucliSENS® easyMAG kit on EMAG device (bioMérieux, Marcy-l’Étoile, France). Sequencing was performed with the Illumina® COVIDSeq Test (Illumina, San Diego, California), which uses 98-target multiplex amplifications along the full SARS-CoV-2 genome. The libraries were sequenced with NextSeq 500/550 High Output Kit v2.5 (75 Cycles) on a NextSeq 500 device (Illumina). The sequences were demultiplexed and assembled as full-length genomes by means of the DRAGEN COVIDSeq Test Pipeline on a local DRAGEN server (Illumina). Lineages and clades were interpreted using Pangolin and NextClade, before being submitted to the GISAID international database (https://www.gisaid.org).

### Statistical analyses

Descriptive results are presented as means (± standard deviation [SD]) or medians (1st–3rd quartiles) for continuous variables, and as numbers with percentages for categorical variables. Two-tailed p values < 0.05 were considered statistically significant. Unadjusted comparisons according to CAPA status (CAPA patients vs. non CAPA patients) were performed using Chi-square or Fisher’s exact tests for categorical variables, and t-tests or Mann–Whitney tests for continuous variables, as appropriate.

Multivariable logistic regression models were performed to identify the parameters most associated with CAPA, entering variables associated with a p-value < 0.20 in univariable analysis and those previously shown to be potential confounding factors, including age and gender, then applying a stepwise backward approach by retaining only variables statistically significant at a relaxed p < 0.10 level. Adjusted odds ratios (aORs) along with their 95% confidence intervals (CI) were computed.

To assess the potential effect of CAPA occurrence on subsequent prognosis, 90-day overall survival was estimated using the Simon–Makuch method [13] and was compared using the Mantel–Byar test between those patients having developed a CAPA and those who had not, considering CAPA occurrence as a time-dependent variable. Cox proportional hazards regression modelling was used to compute Hazard ratios (HR) and their corresponding CIs, with CAPA as a time-dependent covariate and further adjusting for important prognostic factors (i.e., age, gender, baseline SOFA score and immunosuppressive status). Landmark analyses of 90-day overall survival by CAPA status were also conducted as sensitivity analyses. To do so, participants who died or were censored before a 5-days landmark point (as the median time of CAPA occurrence) were excluded from the landmark analyses at day 5 of, allowing a better control of the so called ‘immortal-time bias’ (i.e., patients dying early in the study have a limited time to develop CAPA thus guaranteeing poorer outcomes in the patients unexposed to CAPA) and yielding potentially more accurate results by increasing the number of CAPA patients at risk when starting at a 5-day time point compared to earlier time points when the number of CAPA patients at risk are usually smaller.

An exploratory unsupervised clustering analysis was achieved allowing to explore the heterogeneity of the population using the Kohonen’s self-organized map (SOM) methodology [14], allowing us to build 2-dimensional maps from multidimensional datasets. In a nutshell, each map is divided into districts in which patients are located by the SOM algorithm on the basis of their characteristics: patients with similar features are closely located on the maps, while patients with distinct profiles are farther from each other, hence allowing to identify key differences or similarities among them by drawing visual comparisons of unique or overlapping patient characteristics and disease subtypes. Clinical or biological variables considered as relevant were included in this analysis. The SOMs were obtained with the Numero package framework for the R statistical platform [15] after principal component analysis adapted for mixtures of qualitative and quantitative variables was applied (PCAMix) [16, 17].

All measurements were taken from distinct samples. Variables with missing information used for the evaluation of the risk factors of CAPA by logistic regression, for the evaluation of the effect of CAPA occurrence on overall survival by Cox proportional hazards regression modelling and for the exploratory clustering analysis by self-organizing maps, were imputed using the k-nearest neighbors (k-NN) approach. Analyses were performed using Stata V16.1 statistical software (StataCorp, College Station, TX, USA), and R 4.2.0 (R Foundation for Statistical Computing, Vienna, Austria).

## Results

### Population

**Fig. 1:**
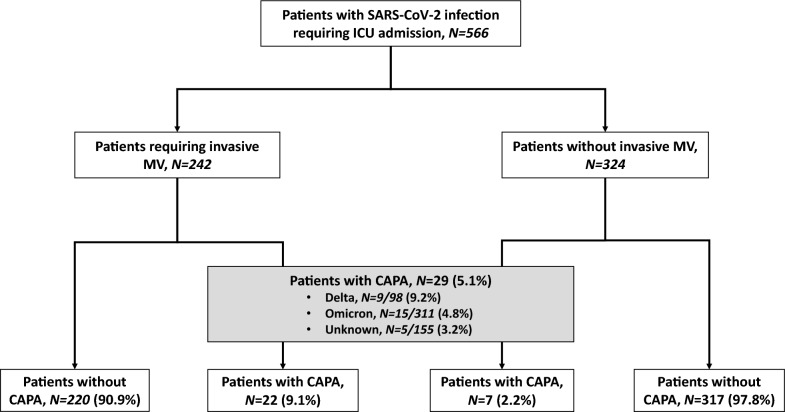
Study flow chart. *CAPA* COVID-19-associated pulmonary aspergillosis; *ICU* intensive care unit

Over the study period, a total of 566 patients were admitted in one of the 36 participating ICUs and included in the study, including 242 patients requiring IMV during ICU stay. Twenty-nine patients (5.1% [95% CI 3.4–7.3]) fulfilled the diagnosis criteria for CAPA (Fig. 1). The prevalence of CAPA was higher in patients who required IMV (*N* = 22/242, 9.1%) than in those who did not (*N* = 7/324, 2.2%).

### Baseline characteristics

Compared to non-CAPA patients, those who fulfilled CAPA diagnosis criteria, did not show statistically significant differences regarding age, gender and comorbidities, except for more frequent immunosuppression (*N* = 15/29, 54% vs. *N* = 174/537, 34%, p = 0.03) (Table 1). Although the proportion of vaccinated patients did not significantly differ according to CAPA status, CAPA patients had more frequently negative anti-S SARS-CoV-2 IgG antibodies than non-CAPA patients, possibly linked to the higher proportion of immunosuppressed patients in the former than in the latter group. The median delay between the first symptoms of disease and ICU admission was significantly longer in CAPA than in non-CAPA patients (8 [6–11] vs. 7 [3–10] days; p = 0.03). The viral load of SARS-CoV-2 in the upper respiratory tract (estimated with the cycle threshold of RT-PCR) did not significantly differ between groups (Table 1). There was no significant difference according to CAPA status regarding the severity of the disease at ICU admission, as reflected by the SOFA and SAPS II scores and the WHO 10-point ordinal scale (Table 1).

**Table 1:** Patient’s characteristics at the time of their admission to the intensive care unit according to the CAPA status

Variable	n/n^a^	All patients, n = 566	Non-CAPA patients, n = 537	CAPA patients, n = 29	p
Demographics and comorbidities					
Sex, females		191 (34)	183 (34)	5 (28)	0.5
Age, years		66 [57–74]	66 [57–74]	67 [60–70]	0.7
Diabetes		179 (33)	168 (33)	11 (39)	0.5
Obesity^b^		183 (33)	172 (32)	11 (38)	0.5
Chronic heart failure		52 (10)	52 (10)	0	0.1
Hypertension		281 (52)	266 (52)	15 (54)	0.8
Chronic respiratory failure		78 (14)	75 (15)	3 (11)	0.8
Chronic renal failure		113 (21)	107 (21)	6 (21)	0.9
Cirrhosis		8 (1)	7 (1)	1 (4)	0.3
Immunosuppression		189 (35)	174 (34)	15 (54)	**0.03**
None		357 (65)	344 (66)	13 (46)	0.09
Solid organ transplant		67 (12)	63 (12)	4 (14)	
Onco-hematological malignancies		59 (11)	54 (10)	5 (18)	
Others^c^		62 (11)	56 (11)	6 (21)	
Number of comorbidities	518/28	2 [1–3]	2 [1–3]	2 [1–4]	0.5
Clinical frailty scale	528/29	3 [2–4]	3 [2–4]	3 [2–4]	0.9
SARS-CoV-2 infection and vaccination					
Previous SARS-CoV-2 infection	506/28	40 (7)	39 (8)	1 (4)	0.8
SARS-CoV-2 vaccination		326 (59)	306 (59)	20 (69)	0.3
SARS-CoV-2 serology at ICU admission					
Unavailable		279 (49)	271 (50)	8 (28)	**0.04**
Negative^d^		129 (23)	119 (22)	10 (34)	
Positive		158 (28)	147 (27)	11 (38)	
First symptoms-ICU admission, days	535/29	7 [3–10]	7 [3–10]	8 [6–11]	**0.03**
SARS-CoV-2 RNA detection in nasopharyngeal swabs, Ct	359/17	21 [18–25]	21 [18–25]	23 [20–26]	0.2
SARS-CoV-2 variant	387/24				
Omicron		313 (76.2)	298 (77)	15 (62.5)	0.1
Delta		98 (23.8)	89 (23)	9 (37.5)	
Patients severity upon ICU admission and biological features					
WHO 10-point scale	353/29	6 [6–6]	6 [6–6]	6 [6–8]	0.09
SAPS II score	486/28	35 [27–45]	35 [27–44]	39 [26–53]	0.1
SOFA score	505/28	4 [3–6]	4 [3–6]	4 [3–8]	0.3
PaO2/FiO2 ratio, mmHg	520/28	124 [79–188]	124 [79–190]	127 [76–170]	0.5
Arterial lactate level, mM	506/27	1.5 [1–2.2]	1.5 [1–2.3]	1.9 [1.1–2.2]	0.6
Blood leukocytes, G/L	529/29	8.9 [5.6–13]	8.9 [5.7–13]	3.9 [6.5–12.4]	0.2
Blood lymphocytes, G/L	434/26	0.5 [0.3–0.9]	0.5 [0.3–0.9]	0.4 [0.5–0.9]	0.9
Blood platelets, G/L	529/29	206 [146–298]	207 [148–289]	191 [107–315]	0.5
Serum urea level, mM	523/29	8 [6–15]	5 [5–14]	12 [7–18]	0.06
Serum creatinine level, µM	532/29	89 [63–141]	89 [62–138]	97 [73–235]	0.1
Lung parenchyma involvement, %	274/18	50 [37–75]	50 [37–75]	50 [40–70]	1
Oxygen/ventilatory support					0.2
Oxygen		100 (18)	97 (18)	3 (10)	
High flow oxygen		269 (48)	255 (48)	14 (48)	
NIV/C-PAP		58 (10)	57 (11)	1 (3)	
Invasive MV		135 (24)	124 (23)	11 (38)	
ECMO		15 (3)	15 (3)	0	1
Vasopressor support		86 (15)	82 (16)	4 (14)	0.8

Results are N (%), means (± standard deviation) or medians (interquartile range, i.e., quartile 1; quartile 3)

*CAPA* COVID-19-associated pulmonary aspergillosis, *ICU* intensive care unit, *Ct* cycle threshold, *WHO* World Health Organization, *SOFA* Sequential Organ Failure Assessment, *SAPS II* Simplified Acute Physiology Score II, *NIV* non-invasive ventilation, *C-PAP* continuous-positive airway pressure, *MV* mechanical ventilation, *ECMO* extracorporeal mechanical ventilation

^a^Numbers of non-CAPA/CAPA patients data available

^b^Defined as a body mass index ≥ 30 kg/m^2^

^c^Includes HIV infection, long-term corticosteroid treatment, and other immunosuppressive treatments

^d^Defined as < 30 Binding Antibody Units (BAU)/mL

Two-tailed p-values come from unadjusted comparisons using Chi-square or Fisher’s exact tests for categorical variables, and t-tests or Mann–Whitney tests for continuous variables, as appropriate. No adjustment for multiple comparisons was performed. Bolded p-values are significant at the p < 0.05 level

CAPA patients tended to require more frequent IMV support within 24 h of ICU admission (*N* = 11/29, 38% vs. *N* = 124/537, 23%, p = 0.2). All three patients requiring extracorporeal membrane oxygenation support upon ICU admission were non-CAPA patients.

### ICU management and outcomes

During ICU stay, CAPA patients required significantly more IMV support (*N* = 22/29, 76% vs. *N* = 220/537, 41%, p = 0.0002) and prone positioning (*N* = 18/29, 64% vs. *N* = 153/537, 30%, p = 0.0002) than their counterparts (Table 2). Of note, IMV duration was significantly longer in CAPA than in non-CAPA patients (28 [17–34] vs. 10 [5–20] days, p = 0.0001) and CAPA patients had more frequent ventilator-acquired pneumonia (VAP) episodes. Median time between start of IMV and the first episode of VAP was 6 [2–10] days globally, and 11 [6–20] days in those patients with CAPA. There were also significant differences between groups regarding need for other organ supports: CAPA patients required significantly more vasopressors and renal replacement therapy. Regarding COVID-19 specific management, there were no significant between-group differences with 83% of patients (*N* = 415/566) who received dexamethasone, 33% (*N* = 165/566) tocilizumab, and 15% (*N* = 74/566) monoclonal antibodies. There was no difference in day-28 mortality according to CAPA status (*N* = 10/29, 34% vs. *N* = 151/537, 29%, p = 0.5), but duration of ICU stay was significantly longer in CAPA patients (28 [16–44] vs. 8 [4–17] days, p < 0.0001).

**Table 2:** Management and outcomes of critically ill COVID-19 patients (n = 566) during their intensive care unit stay according to Coronavirus disease (COVID-19)-associated pulmonary aspergillosis (CAPA) status

Variable		n/n^b^	All patients, n = 566	Non-CAPA patients, n = 537	CAPA patients, n = 29	p
Invasive MV			242 (43)	220 (41)	22 (76)	**0.0002**
Prone positioning			171 (32)	153 (30)	18 (64)	**0.0002**
MV duration, days		207/21	12 [5–22]	10 [5–20]	28 [17–34]	**0.0001**
Ventilator-free days at D28			25 [0–28]	26 [0–28]	0 [0–15]	**0.0004**
ECMO support			32 (6)	29 (5)	3 (10)	0.2
Duration of ECMO, days		25/3	27 [10–55]	29 [10–62]	19 [15–20]	0.4
Vasopressor support			218 (39)	197 (37)	21 (72)	**0.0003**
Duration of vasopressors, days		192/20	27 [10–55]	29 [10–62]	19 [15–20]	0.4
Renal replacement therapy			69 (12)	60 (11)	9 (31)	**0.001**
Ventilator-acquired pneumonia (among IMV)^a^			126 (52)	108 (49)	18 (82)	**0.003**
Time from IMV to VAP first episode, days			6 [2–10]	6 [2–9]	11 [6–20]	**0.003**
Number of VAP episodes	Median (IQR)		1 [0–1]	1 [0–1]	1 [1, 2]	**0.007**
	0		116 (48)	112 (51)	4 (18)	**0.01**
	1		66 (27)	56 (26)	10 (45)	
	2		40 (17)	35 (16)	5 (23)	
	3		19 (8)	16 (7)	3 (14)	
Dexamethasone			415 (83)	392 (83)	23 (82)	0.9
Tocilizumab			165 (33)	153 (33)	9 (33)	0.9
Monoclonal antibodies			74 (15)	67 (14)	7 (25)	0.1
Day-28 mortality			161 (29)	151 (29)	10 (34)	0.5
Duration of ICU stay, days		522/29	9 [4–18]	8 [4–17]	28 [16–44]	** < 0.0001**

Results are *N* (%), means (± standard deviation) or medians (interquartile range, i.e., quartile 1; quartile 3)

*CAPA,* COVID-19-associated pulmonary aspergillosis, *MV* mechanical ventilation, *ECMO,* extracorporeal membrane oxygenation, *VAP,* ventilator-acquired pneumonia, *IMV,* invasive mechanical ventilation

^a^VAP episodes were recorded per definition in patients under IMV since more than 48 h

^b^Numbers of non-CAPA/CAPA patients data available

Two-tailed *p*-values come from unadjusted comparisons using Chi-square or Fisher’s exact tests for categorical variables, and *t*-tests or Mann–Whitney tests for continuous variables, as appropriate. No adjustment for multiple comparisons was performed. Bolded p-values are significant at the p < 0.05 level

**Fig. 2:**
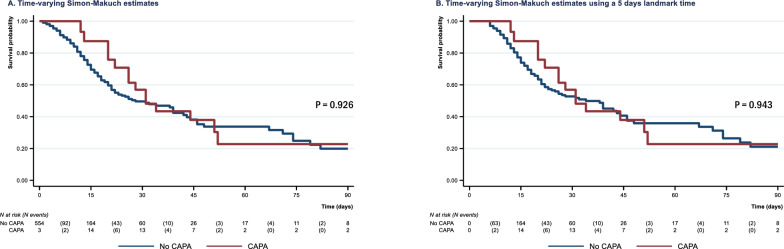
**a** Simon–Makuch estimates of overall survival status from ICU admission to day-90 according to CAPA status; **b** Simon–Makuch estimates of overall survival status from ICU admission to day-90 according to CAPA status using landmark times at 5 days

The Simon-Makuch estimates of overall survival from ICU admission to day-90 is depicted in Fig. 2. No statistically significant association was found between CAPA occurrence and mortality, whether considering Mantel-Byar test (p = 0.926) or Cox time-dependent analyses (HR 0.97 (0.56–1.70), p = 0.927; adjusted HR after missing data imputation 0.79 (0.45–1.41), p = 0.425; adjusted HR on raw data 0.76 (0.42–1.40), p = 0.382). Sensitivity analyses considering a 5-day timepoint yielded similar results (Mantel-Byar p = 0.943; HR 1.02 [0.58–1.79], p = 0.944; adjusted HR after missing data imputation 0.90 [0.51–1.60], p = 0.719; adjusted HR on raw data 0.83 [0.45–1.53], p = 0.551).

### Characteristics of CAPA patients

**Fig. 3:**
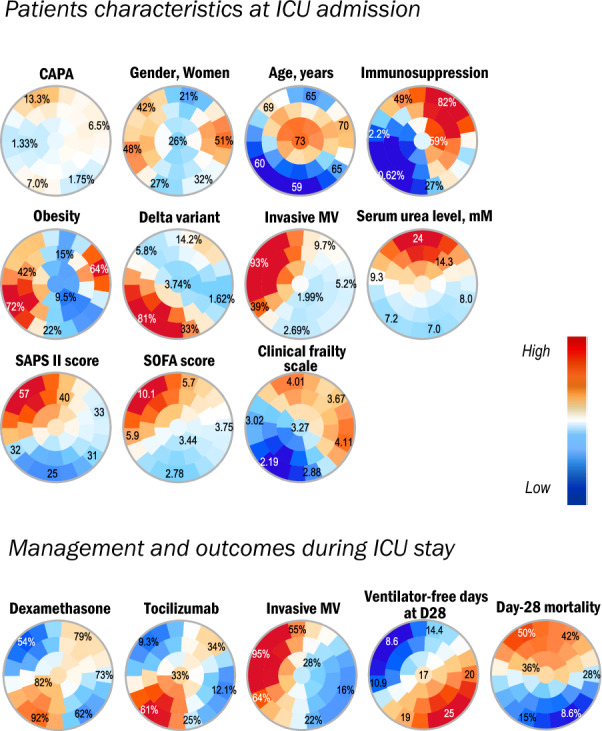
Unsupervised analysis of the clinical and biological characteristics of the by self-organized maps (SOMs). Unsupervised analysis by SOM automatically located patients with similar clinical and paraclinical parameters within 1 of 40 small groupings (“districts”) throughout the map. The more similar the patients, the closer on the map. Each individual map shows the mean values or proportions per district for each characteristic: blue indicates the lowest average values, red the highest, with numbers shown for a selection of representative districts in each SOM. For instance, immunosuppressed patients were more frequently located in the upper districts and also had higher serum urea levels, less frequent Delta variant infection, higher SAPS II and SOFA scores and day-28 mortality rates. WHO World Health Organization, SOFA Sequential Organ Failure Assessment, SAPS II Simplified Acute Physiology Score II, MV mechanical ventilation

According to CMM/ISHAM CAPA definitions, 24 patients (83%) fulfilled the criteria of proven/probable CAPA and 5 patients (17%) were classified as possible CAPA (Table 3). The detailed diagnostic criteria are shown in Additional file 1: Figure S1. The diagnosis of CAPA was made a median of 5 [2–16] days after ICU admission and 6 [2–13] days after tracheal intubation (among the CAPA patients requiring IMV). Half of the patients were immunosuppressed: 10 (66%) had an onco-hematological malignancy, and 4 (27%) had received an organ transplant. Among the 29 CAPA patients, 28 (97%) received an antifungal treatment during ICU stay. Voriconazole and isavuconazole were the two most frequently administered antifungal drugs (Table 3). To better characterize the phenotypic differences between CAPA and non-CAPA patients, an exploratory analysis using the SOM method was performed to plot 2-D maps of patients grouped according to their characteristics (Fig. 3). SOM analysis depicted the observed differences according to CAPA status. As shown in the figure, CAPA patients tended to cluster in the upper left area of the map, where the highest frequencies of immunosuppression, the highest values of the SOFA and SAPS II scores also clustered. Patients with the highest rates of day-28 mortality and use of IMV during ICU stay clustered in the same area of the map than CAPA patients.

**Table 3:** Diagnosis criteria, characteristics and antifungal therapy of patients (n = 29) diagnosed with Coronavirus disease (COVID-19)-associated pulmonary aspergillosis (CAPA)

Variable	CAPA patients, n = 29
CAPA diagnosis criteria	
CAPA proven/probable	24 (83)
CAPA possible	5 (17)
Time from ICU admission to CAPA diagnosis, days	5 [2–16]
Immunosuppression	15 (52)
Haematological malignancies	10 (66)
Solid organ transplantation	4 (27)
Immunosuppressive drugs	1 (7)
IMV	22 (76)
Time from IMV to CAPA diagnosis, days	6 [2–13]
Antifungal therapy	28 (97)
Azole antifungals	28 (100)
Voriconazole	24 (86)
Isavuconazole	4 (14)
Itraconazole	1 (4)
Posaconazole	1 (4)
Caspofungin	2 (7)
Liposomal amphotericin B	2 (7)

Results are *N* (%), means (± standard deviation) or medians (interquartile range, i.e., quartile 1; quartile 3)

*CAPA* COVID-19-associated pulmonary aspergillosis, *ICU* intensive care unit, *IMV* invasive mechanical ventilation

### Factors associated with CAPA

In multivariable analysis after missing data imputation, four factors associated with higher risk of CAPA were retained after stepwise analysis: increased SAPS II score (aOR 1.03 [95% CI 1.003–1.05], p = 0.028), immunosuppression (aOR 2.65 [1.13–6.20], p = 0.025), a SARS-CoV-2 Delta variant (aOR 2.72 [1.12–6.58], p = 0.027), and to a lower extent an increased delay between the first symptoms and ICU admission (aOR 1.03 [0.997–1.06], p = 0.077) (Table 4). Age and IMV support during ICU stay were not significantly associated with CAPA at the p < 0.10 level. Results of the multivariable analysis on raw data are presented in Additional file 3: Table S2.

**Table 4:** Predictors of CAPA occurrence by univariable and multivariable logistic regression models in critically ill patients with COVID-19: results after missing data imputation (n = 566)

Factor	Univariable Analysis		p-value	Multivariable Analysis		
	OR	95% CI		aOR	95% CI	p-value
Blood leukocytes, G/L	1.02	(1.004;1.05)	**0.018**			
SAPS II score	1.02	(1.001;1.05)	**0.047**	1.03	(1.003;1.05)	**0.028**
Age, years	1.00	(0.98;1.03)	0.740			
Gender, females	0.74	(0.32;1.70)	0.473			
SOFA score	1.13	(1.01;1.26)	**0.028**			
Serum urea level, mM	1.01	(0.99;1.03)	0.368			
First symptoms—ICU admission, days	1.03	(1.001;1.06)	**0.042**	1.03	(0.997;1.06)	0.077
Invasive mechanical ventilation	2.04	(0.94;4.42)	0.073			
Immunosuppression	2.36	(1.116;5.01)	**0.025**	2.65	(1.13;6.20)	**0.025**
SARS-CoV-2 variant						
Omicron (ref)	1 (ref)			1 (ref)		
Delta	1.63	(0.74;3.60)	0.225	2.72	(1.12;6.58)	**0.027**

aOR (CI 95%): adjusted Odds Ratio (95% confidence interval)

*CAPA* COVID-19-associated pulmonary aspergillosis, *ICU* intensive care unit, *SAPS* simplified acute physiology score; *SOFA* Sequential Organ Failure Assessment

p-values come from multivariable logistic regression models

Bolded p-values are significant at the p < 0.05 level

## Discussion

To the best of our knowledge, we herein report the largest cohort study investigating the prevalence and the characteristics of CAPA among critically-ill COVID-19 patients in the era of Delta and Omicron SARS-CoV-2 variants. The main results of our study are the following: (i) the prevalence of CAPA in the whole cohort was 5.1%, and 9.1% among patients requiring IMV; (ii) CAPA patients were more frequently immunosuppressed, required more frequently IMV, vasopressors and renal replacement therapy during ICU stay; (iii) CAPA was not associated with day-28 mortality, but with a longer duration of mechanical ventilation support and ICU stay; and (iv) SAPS II score at ICU admission and the delay between the first symptoms and ICU admission were independently associated with CAPA.

The prevalence of CAPA herein reported (5.1%) is lower than previously reported in studies conducted during the first wave in mechanically ventilated ICU patients: 11–28% [1, 6, 18, 19] or in more recent studies: 16–33% [20, 21]. Several factors may explain these results. First, these studies were carried out only in mechanically ventilated patients, while more than half of the patients in our cohort did not require IMV. Indeed, in the subgroup of patients requiring IMV, the reported prevalence was higher (9.1%). Second, these studies routinely screened for diagnosis criteria for CAPA once or twice a week, whereas it was at the initiative of the attending clinician in our study (i.e., targeted sampling). Whether bronchoalveolar lavage sampling should be routinely performed to increase the sensitivity of the diagnosis criteria has been suggested [22]. We acknowledge that our study may be associated with a lower prevalence of CAPA than that reported in other studies performing routine screening [20], however our targeted sampling strategy reflects real-life practice in a nation-wide study. The most appropriate diagnostic strategy for CAPA remains to be defined. Indeed, as most of the CAPA diagnostic criteria are nonspecific (i.e., biomarkers, non-specific clinical and/or radiological signs), a systematic diagnostic approach (as opposed to targeted sampling) might be associated with a lower pre-test probability and thus a lower positive predictive value of CAPA diagnosis. Third and importantly, the clinical phenotype of critically-ill COVID-19 patients has evolved in line with the natural course of the disease, with older and frailer patients, more frequently immunosuppressed, and presenting with higher severity scores at ICU admission [7]. Yet, in the current series, the prevalence of CAPA among patients infected with Omicron (4.8%) was lower than that observed in patients infected with Delta (9.2%), and lower than that reported during the first pandemic wave [1]. These findings might point to a variant-related effect. CAPA has been associated with impaired antifungal immunity (i.e., altered integrity of the epithelial barrier, and decreased capacity to phagocytise and kill Aspergillus spores and to destroy Aspergillus hyphae) [23]. Recent findings suggested a reduced evasion of variant Omicron from innate immunity [24, 25], as compared to pre-existing variants, which might be associated with more effective antifungal immunity and hence a lower prevalence of CAPA. Another potential factor might be the inherent disorganization during the first epidemic wave, resulting in less frequent use of rooms with negative pressure in comparison with the period of this study (i.e., Delta and Omicron era).

We also describe the existence of CAPA in non-intubated patients, although occurring in only 2.2% of patients, which had rarely been reported to date. Indeed, previous studies investigating CAPA included almost exclusively patients requiring IMV [1, 18, 20, 21, 26]. However, two multicenter studies reported that a minority of CAPA occurred in non-intubated patients [6, 27], 6% and 12% of CAPA patients, respectively. Unfortunately, their design did not allow for a reliable assessment of the prevalence of CAPA in this setting. In our study, however, most of non-intubated CAPA patients had a specific underlying condition (five patients were immunocompromised (four haematological malignancies and one solid organ transplantation) and one patient had a chronic cavitary pulmonary aspergillosis, rather than a “classical” CAPA. No classical risk factor was identified for the last patient. Therefore, it appears appropriate to screen non-intubated COVID-19 patients for CAPA in case of underlying immunosuppressive status or other risk factors, rather than routinely.

Previous studies identified various factors associated with CAPA: age, long-term corticosteroids use, chronic obstructive pulmonary disease, haematological malignancy, IMV and its prolonged duration, tocilizumab treatment, especially in association with corticosteroids [1, 6, 18, 19, 28–31]. In our study, we could identify three factors independently associated with CAPA in multivariable analysis, including SAPS II score at admission, immunosuppression and a SARS-CoV-2 Delta variant. The first two factors are thus in line with previous findings; whereas the Delta variant effect has not yet been described [20]. It is important to note most of the previous studies included patients before the Omicron era, and our study is the largest one investigating CAPA in the era of Omicron.

We did not observe an association between day-28 mortality and CAPA status in our study, in contrast with previous studies that reported a high mortality associated with CAPA [1, 6, 18, 19, 26, 32]. Nevertheless, duration of mechanical ventilation and ICU stay were significantly longer in CAPA patients than non-CAPA patients. Moreover, vital status was only reported at day-28, and it is likely that CAPA status was associated with longer-term prognosis. In line with previous studies, the diagnosis of CAPA was mostly reliant on serum and/or respiratory fungal markers. Thus, the prognostic relevance of CAPA per se could be questioned: it might be considered a simple severity indicator rather than a truly invasive super-infection. A recent pathology-based studies has highlighted the invasive nature of CAPA [5], thereby supporting systematic CAPA screening of intubated patients and specific antifungal treatment.

Our study has some limitations. We were unable to compare these results with those from the first waves, as this prospective cohort began during Delta variant era. The relatively small number of CAPA patients included may have limited our statistical ability to show between-group differences. Non-systematic screening for CAPA may have underestimated the prevalence of CAPA. However, our study also has strengths, including the constitution of a unique prospective multicenter cohort reporting the prevalence and characteristics of CAPA in the era of Delta and Omicron SARS-CoV-2 variants.

## Conclusion

To conclude, we report a lower prevalence of CAPA (5.1%) among critically-ill COVID-19 patients in the era of Delta and Omicron variants than previously reported, and mainly affecting intubated-patients. SAPS II score at ICU admission, immunosuppression status and a SARS-CoV-2 Delta variant were independently associated with CAPA. Even though CAPA was not associated with day-28 mortality, duration of mechanical ventilation and ICU stay were significantly longer in CAPA patients.

## Supplementary Information

**Additional file 1: Figure S1.** Diagnosis criteria of CAPA (proven/probable and possible), relied on ECMM/ISHAM consensus criteria. BAL, bronchoalveolar lavage; CAPA, COVID-19-associated pulmonary aspergillosis; PCR, polymerase chain reaction.

**Additional file 2: Table S1.** Criteria used for the classification of patients according to the ECMM/ISHAM consensus criteria.

**Additional file 3: Table S2.** Predictors of CAPA occurrence by univariable and multivariable logistic regression models in critically ill patients with COVID-19: results on raw data (n = 566).

## Data Availability

The clinical datasets generated during and/or analyzed during the current study are available from the corresponding author on reasonable request (N.D.P.)

## Abbreviations

ARDS: Acute respiratory distress syndrome
CAPA: COVID-19-associated pulmonary aspergillosis
IAPA: Influenza-associated pulmonary aspergillosis
ICU: Intensive care unit
IMV: Invasive mechanical ventilation
HR: Hazard ratio
SAPS: Simplified acute physiology score
SOFA: Sequential organ failure assessment
VAP: Ventilator-associated pneumonia
WHO: World Health Organization
