# Correction: COVID-19 associated pulmonary aspergillosis in critically-ill patients: a prospective multicenter study in the era of Delta and Omicron variants

**DOI:** 10.1186/s13613-024-01318-x

**Published:** 2024-06-14

**Authors:** Pierre Bay, Etienne Audureau, Sébastien Préau, Raphaël Favory, Aurélie Guigon, Nicholas Heming, Elyanne Gault, Tài Pham, Amal Chaghouri, Matthieu Turpin, Laurence Morand-Joubert, Sébastien Jochmans, Aurélia Pitsch, Sylvie Meireles, Damien Contou, Amandine Henry, Adrien Joseph, Marie-Laure Chaix, Fabrice Uhel, Damien Roux, Diane Descamps, Malo Emery, Claudio Garcia-Sanchez, David Levy, Sonia Burrel, Julien Mayaux, Antoine Kimmoun, Cédric Hartard, Frédéric Pène, Flore Rozenberg, Stéphane Gaudry, Ségolène Brichler, Antoine Guillon, Lynda Handala, Fabienne Tamion, Alice Moisan, Thomas Daix, Sébastien Hantz, Flora Delamaire, Vincent Thibault, Bertrand Souweine, Cecile Henquell, Lucile Picard, Françoise Botterel, Christophe Rodriguez, Armand Mekontso Dessap, Jean-Michel Pawlotsky, Slim Fourati, Nicolas de Prost, the SEVARVIR investigators, Keyvan Razazi, Raphaël Bellaïche, Elie Azoulay, Jean-François Timsit, Guillaume Voiriot, Nina de Montmollin, Stéphane Marot, Maxime Gasperment, Tomas Urbina, Hafid Ait Oufella, Eric Maury Djeneba Bocar Fofana, Charles-Edouard Luyt, Djillali Annane, Ferhat Meziani, Louis-Marie Jandeaux, Samira Fafi-Kremer, Cédric Darreau, Jean Thomin, Anaïs Dartevel, Sylvie Larrat, Evelyne Schvoerer, Béatrice La Combe, Séverine Haouisee, Sami Hraeich, Pierre-Edouard Fournier, Philippe Colson, Emmanuel Canet, Berthe Marie Imbert, Guillaume Thiery, Sylvie Pillet, Rémy Coudroy, Nicolas Leveque, Clément Saccheri, Valérie Giordanengo, Kada Klouche, Edouard Tuaillon, Cécile Aubron, Adissa Tran, Jean-Marc Tadié, Jean-Christophe Plantier, Sophie Vallet

**Affiliations:** 1 Médecine Intensive Réanimation, Hôpitaux Universitaires Henri Mondor, Assistance Publique-Hôpitaux de Paris (AP-HP), CHU Henri Mondor, 51, Av. de Lattre de Tassigny, CEDEX, 94010 Créteil, France; 2 Groupe de Recherche Clinique CARMAS, Université Paris-Est-Créteil (UPEC), Créteil, France; 3 Université Paris-Est-Créteil (UPEC), Créteil, France; 4 IMRB INSERM U955, Team “Viruses, Hepatology, Cancer”, Créteil, France; 5 IMRB INSERM U955, Team CEpiA, Créteil, France; 6 Unité de Recherche Clinique, Department of Public Health, Hôpitaux Universitaires Henri Mondor, Assistance Publique-Hôpitaux de Paris (AP-HP), Créteil, France; 7 U1167-RID-AGE Facteurs de Risque et Déterminants Moléculaires des Maladies Liées au Vieillissement, University Lille, Inserm, CHU Lille, Institut Pasteur de Lille, 59000 Lille, France; 8 Service de Virologie, CHU de Lille, 59000 Lille, France; 9 Médecine Intensive Réanimation, Hôpital Raymond Poincaré, Assistance Publique-Hôpitaux de Paris (AP-HP), Garches, France; 10 Laboratoire de Virologie, Hôpital Ambroise Paré, Assistance Publique-Hôpitaux de Paris (AP-HP), Boulogne, France; 11 Service de Médecine Intensive-Réanimation, Assistance Publique-Hôpitaux de Paris, Hôpital de Bicêtre, DMU 4 CORREVE Maladies du Cœur et des Vaisseaux, FHU Sepsis, Le Kremlin-Bicêtre, France; 12 Inserm U1018, Equipe d’Epidémiologie Respiratoire Intégrative, CESP, 94807 Villejuif, France; 13 Laboratoire de Virologie, Hôpital Paul Brousse, Assistance Publique-Hôpitaux de Paris, Villejuif, France; 14 Centre de Recherche Saint-Antoine INSERM, Médecine Intensive Réanimation, Hôpital Tenon, Assistance Publique-Hôpitaux de Paris, Sorbonne Université, Paris, France; 15 INSERM, Institut Pierre Louis d’Epidémiologie et de Santé Publique, Sorbonne Université, Paris, France; 16 Laboratoire de Virologie, Hôpital Saint-Antoine, Assistance Publique-Hôpitaux de Paris, 75012 Paris, France; 17 Service de Réanimation Polyvalente, Hôpital Marc Jacquet, Melun, France; 18 Laboratoire de Microbiologie, Hôpital Marc Jacquet, Melun, France; 19 Service de Réanimation Médico-Chirurgicale, Assistance Publique-Hôpitaux de Paris, Hôpital Ambroise Paré, Boulogne, France; 20 Service de Réanimation, Hôpital Victor Dupouy, Argenteuil, France; 21 Service de Virologie, Hôpital Victor Dupouy, Argenteuil, France; 22 Médecine Intensive Réanimation, Hôpital Saint-Louis, Assistance Publique-Hôpitaux de Paris, Paris, France; 23 Inserm HIPI, Université Paris Cité, 75010 Paris, France; 24 Laboratoire de Virologie, Hôpital Saint-Louis, Assistance Publique-Hôpitaux de Paris, 75010 Paris, France; 25 DMU ESPRIT, Service de Médecine Intensive Réanimation, Université Paris Cité, APHP, Hôpital Louis Mourier, Colombes, France; 26 INSERM U1151, CNRS UMR 8253, Department of Immunology, Infectiology and Hematology, Institut Necker-Enfants Malades (INEM), Paris, France; 27 IAME INSERM UMR 1137, Service de Virologie, Hôpital Bichat-Claude Bernard, Assistance Publique-Hôpitaux de Paris, Université Paris Cité, Paris, France; 28 Service de Réanimation, Hôpital Saint-Camille, Bry-Sur-Marne, France; 29 Laboratoire de Biologie, Hôpital Saint-Camille, Bry-Sur-Marne, France; 30 Assistance Publique-Hôpitaux de Paris, Hôpital Pitié-Salpêtrière, Réanimation Médicale, Sorbonne Université, Paris, France; 31 Service de Virologie, CHU de Bordeaux et CNRS UMR 5234, Fundamental Microbiology and Pathogenicity, Université de Bordeaux, Bordeaux, France; 32 Département de Virologie, Hôpital Pitié-Salpêtrière, Assistance Publique-Hôpitaux de Paris (AP-HP), Paris, France; 33 Assistance Publique-Hôpitaux de Paris, Hôpital Pitié-Salpêtrière, Médecine Intensive Réanimation, Sorbonne Université, Paris, France; 34 CHRU de Nancy, Médecine Intensive et Réanimation Brabois, Université de Lorraine, Vandœuvre-Lès-Nancy, France; 35 INSERM U942 and U1116, F-CRIN-INIC RCT, Vandœuvre-Lès-Nancy, France; 36 Service de Virologie, CHRU de Nancy, Vandœuvre-Lès-Nancy, France; 37 Médecine Intensive Réanimation, Hôpital Cochin, Assistance Publique-Hôpitaux de Paris, Paris, France; 38 Laboratoire de Virologie, Hôpital Cochin, Assistance Publique-Hôpitaux de Paris, Paris, France; 39 Service de Réanimation, Hôpital Avicenne, Assistance Publique-Hôpitaux de Paris, Bobigny, France; 40 Laboratoire de Virologie, Hôpital Avicenne, Assistance Publique-Hôpitaux de Paris, Bobigny, France; 41 Intensive Care Unit, Tours University Hospital, Research Center for Respiratory Diseases (CEPR), INSERM U1100, University of Tours, Tours, France; 42 INSERM U1259, Université de Tours, Tours, France; 43 CHRU de Tours, National Reference Center for HIV-Associated Laboratory, Tours, France; 44 Service de Médecine Intensive-Réanimation, CHU De Rouen, Rouen, France; 45 INSERM, Normandie Univ, DYNAMICURE UMR 1311, CHU Rouen, Department of Virology, Univ Rouen Normandie, Université de Caen Normandie, 76000 Rouen, France; 46 Réanimation Polyvalente, INSERM CIC 1435 and UMR 1092, CHU Limoges, Limoges, France; 47 French National Reference Center for Herpesviruses, Bacteriology, Virology, Hygiene Department, CHU Limoges, 87000 Limoges, France; 48 INSERM, RESINFIT, U1092, 87000, Limoges, France; 49 CHU Rennes, Maladies Infectieuses et Réanimation Médicale, Rennes, France; 50 Laboratoire de Virologie, CHU Rennes, 35000 Rennes, France; 51 Inserm, EHESP, Irset (Institut de Recherche en Santé, Environnement et Travail) UMR_S 1085, Univ Rennes, 35000 Rennes, France; 52 Service de Médecine Intensive et Réanimation, CHU Clermont-Ferrand, Clermont-Ferrand, France; 53 3IHP, Service de Virologie, CHU Clermont-Ferrand, Clermont-Ferrand, France; 54 Département d’Anesthésie Réanimations Chirurgicales, Hôpitaux Universitaires Henri Mondor, Assistance Publique-Hôpitaux de Paris (AP-HP), Créteil, France; 55 Department of Virology, Hôpitaux Universitaires Henri Mondor, Assistance Publique-Hôpitaux de Paris, Créteil, France

**Correction: Annals of Intensive Care (2024) 14:65** 10.1186/s13613-024-01296-0

Following publication of the original article [1], the authors identified an error in Tables 1 and 2.

In Table 1, the data for the “**Sex, females**” under the heading of “**CAPA patients, n = 29”** should be **8 (28)** not 5 (28).

The correct Table 1 is given in this correction.

**Table 1:** Patient’s characteristics at the time of their admission to the intensive care unit according to the CAPA status

Variable	n/n^a^	All patients, n = 566	Non-CAPA patients, n = 537	CAPA patients, n = 29	p
Demographics and comorbidities					
Sex, females		191 (34)	183 (34)	8 (28)	0.5
Age, years		66 [57–74]	66 [57–74]	67 [60–70]	0.7
Diabetes		179 (33)	168 (33)	11 (39)	0.5
Obesity		183 (33)	172 (32)	11 (38)	0.5
Chronic heart failure		52 (10)	52 (10)	0	0.1
Hypertension		281 (52)	266 (52)	15 (54)	0.8
Chronic respiratory failure		78 (14)	75 (15)	3 (11)	0.8
Chronic renal failure		113 (21)	107 (21)	6 (21)	0.9
Cirrhosis		8 (1)	7 (1)	1 (4)	0.3
Immunosuppression		189 (35)	174 (34)	15 (54)	**0.03**
None		357 (65)	344 (66)	13 (46)	0.09
Solid organ transplant		67 (12)	63 (12)	4 (14)	
Onco-hematological malignancies		59 (11)	54 (10)	5 (18)	
Others^b^		62 (11)	56 (11)	6 (21)	
Number of comorbidities	518/28	2 [1–3]	2 [1–3]	2 [1–4]	0.5
Clinical frailty scale	528/29	3 [2–4]	3 [2–4]	3 [2–4]	0.9
SARS-CoV-2 infection and vaccination					
Previous SARS-CoV-2 infection	506/28	40 (7)	39 (8)	1 (4)	0.8
SARS-CoV-2 vaccination		326 (59)	306 (59)	20 (69)	0.3
SARS-CoV-2 serology at ICU admission					
Unavailable		279 (49)	271 (50)	8 (28)	**0.04**
Negative^c^		129 (23)	119 (22)	10 (34)	
Positive		158 (28)	147 (27)	11 (38)	
First symptoms-ICU admission, days	535/29	7 [3–10]	7 [3–10]	8 [6–11]	**0.03**
SARS-CoV-2 RNA detection in nasopharyngeal swabs, Ct	359/17	21 [18–25]	21 [18–25]	23 [20–26]	0.2
SARS-CoV-2 variant	387/24				
Omicron		313 (76.2)	298 (77)	15 (62.5)	0.1
Delta		98 (23.8)	89 (23)	9 (37.5)	
Patients severity upon ICU admission and biological features					
WHO 10-point scale	353/29	6 [6–6]	6 [6–6]	6 [6–8]	0.09
SAPS II score	486/28	35 [27–45]	35 [27–44]	39 [26–53]	0.1
SOFA score	505/28	4 [3–6]	4 [3–6]	4 [3–8]	0.3
PaO_2_/FiO_2_ ratio, mmHg	520/28	124 [79–188]	124 [79–190]	127 [76–170]	0.5
Arterial lactate level, mM	506/27	1.5 [1–2.2]	1.5 [1–2.3]	1.9 [1.1–2.2]	0.6
Blood leukocytes, G/L	529/29	8.9 [5.6–13]	8.9 [5.7–13]	3.9 [6.5–12.4]	0.2
Blood lymphocytes, G/L	434/26	0.5 [0.3–0.9]	0.5 [0.3–0.9]	0.4 [0.5–0.9]	0.9
Blood platelets, G/L	529/29	206 [146–298]	207 [148–289]	191 [107–315]	0.5
Serum urea level, mM	523/29	8 [6–15]	8 [5–14]	12 [7–18]	0.06
Serum creatinine level, µM	532/29	89 [63–141]	89 [62–138]	97 [73–235]	0.1
Lung parenchyma involvement, %	274/18	50 [37–75]	50 [37–75]	50 [40–70]	1
Oxygen/ventilatory support					0.2
Oxygen		100 (18)	97 (18)	3 (10)	
High flow oxygen		269 (48)	255 (48)	14 (48)	
NIV/C-PAP		58 (10)	57 (11)	1 (3)	
Invasive MV		135 (24)	124 (23)	11 (38)	
ECMO		15 (3)	15 (3)	0	1
Vasopressor support		86 (15)	82 (16)	4 (14)	0.8

Results are N (%), means (± standard deviation) or medians (interquartile range, i.e., quartile 1; quartile 3)

*CAPA* COVID-19-associated pulmonary aspergillosis, *ICU* intensive care unit, *Ct* cycle threshold, *WHO* World Health Organization, *SOFA* Sequential Organ Failure Assessment, *SAPS II* Simplified Acute Physiology Score II, *NIV* non-invasive ventilation, *C-PAP* continuous-positive airway pressure, *MV* mechanical ventilation, *ECMO* extracorporeal mechanical ventilation

Two-tailed p-values come from unadjusted comparisons using Chi-square or Fisher’s exact tests for categorical variables, and t-tests or Mann–Whitney tests for continuous variables, as appropriate. No adjustment for multiple comparisons was performed. Bolded p-values are significant at the p < 0.05 level

^a^Numbers of non-CAPA/CAPA patients data available

^b^Includes HIV infection, long-term corticosteroid treatment, and other immunosuppressive treatments

^c^Defined as < 30 Binding Antibody Units (BAU)/mL

In Table 2, the **data for Duration of vasopressors**, days under the heading of variable has been corrected in this correction and the complete Table 2 is given in this correction.

**Table 2:** Management and outcomes of patients with severe SARS-CoV-2 infection during their intensive care unit stay according to the CAPA status

Variable	n/n^a^	All patients, n = 566	Non-CAPA patients, n = 537	CAPA patients, n = 29	p
Invasive MV		242 (43)	220 (41)	22 (76)	**0.0002**
Prone positioning		171 (32)	153 (30)	18 (64)	**0.0002**
MV duration, days	207/21	12 [5–22]	10 [5–20]	28 [17–34]	**0.0001**
Ventilator-free days at D28		25 [0–28]	26 [0–28]	0 [0–15]	**0.0004**
ECMO support		32 (6)	29 (5)	3 (10)	0.2
Duration of ECMO, days	25/3	27 [10–55]	29 [10–62]	19 [15–20]	0.4
Vasopressor support		218 (39)	197 (37)	21 (72)	**0.0003**
Duration of vasopressors, days	192/20	4 [1–12]	4 [1–10]	16 [9–30]	**0.0002**
Renal replacement therapy		69 (12)	60 (11)	9 (31)	**0.001**
Ventilator-acquired pneumonia (among IMV)^b^		126 (52)	108 (49)	18 (82)	**0.003**
Time from IMV to VAP first episode, days		6 [2–10]	6 [2–9]	11 [6–20]	**0.003**
Number of VAP episodes					
Median (IQR)		1 [0–1]	1 [0–1]	1 [1–2]	**0.007**
0		116 (48)	112 (51)	4 (18)	**0.01**
1		66 (27)	56 (26)	10 (45)	
2		40 (17)	35 (16)	5 (23)	
3		19 (8)	16 (7)	3 (14)	
Dexamethasone		415 (83)	392 (83)	23 (82)	0.9
Tocilizumab		165 (33)	156 (33)	9 (33)	0.9
Monoclonal antibodies		74 (15)	67 (14)	7 (25)	0.1
Day-28 mortality		161 (29)	151 (29)	10 (34)	0.5
Duration of ICU stay, days	522/29	9 [4–18]	8 [4–17]	28 [16–44]	**< 0.0001**

Results are *N* (%), means (± standard deviation) or medians (interquartile range, i.e., quartile 1; quartile 3)

*CAPA* COVID-19-associated pulmonary aspergillosis, *MV* mechanical ventilation, *ECMO* extracorporeal membrane oxygenation, *VAP* ventilator-acquired pneumonia, *IMV* invasive mechanical ventilation

Two-tailed *p*-values come from unadjusted comparisons using Chi-square or Fisher’s exact tests for categorical variables, and *t*-tests or Mann–Whitney tests for continuous variables, as appropriate. No adjustment for multiple comparisons was performed. Bolded p-values are significant at the p < 0.05 level

^a^Numbers of non-CAPA/CAPA patients data available

^b^VAP episodes were recorded per definition in patients under IMV since more than 48 h

The correct Table 2 is given in this correction.

In this article, the legend for Fig. 3 was incorrectly published.

Incorrect legend of Fig. 3:

**Fig. 3** Unsupervised analysis of the clinical and biological characteristics of the by self-organized maps (SOMs). Unsupervised analysis by SOM automatically located patients with similar clinical and paraclinical parameters within 1 of 40 small groupings (“districts”) throughout the map. The more similar the patients, the closer on the map. Each individual map shows the mean values or proportions per district for each characteristic: blue indicates the lowest average values, red the highest, with numbers shown for a selection of representative districts in each SOM. For instance, immunosuppressed patients were more frequently located in the upper districts and also had higher serum urea levels, less frequent Delta variant infection, higher SAPS II and SOFA scores and day-28 mortality rates. WHO World Health Organization, SOFA Sequential Organ Failure Assessment, SAPS II Simplifed Acute Physiology Score II, MV mechanical ventilation

Correct legend of Fig. 3:

**Fig. 3** Unsupervised analysis of the clinical and biological characteristics of the 566 critically-ill COVID-19 patients by self-organized maps (SOMs). Unsupervised analysis by SOM automatically located patients with similar clinical and paraclinical parameters within 1 of 40 small groupings (“districts”) throughout the map. The more similar the patients, the closer on the map. Each individual map shows the mean values or proportions per district for each characteristic: blue indicates the lowest average values, red the highest, with numbers shown for a selection of representative districts in each SOM. For instance, immunosuppressed patients were more frequently located in the upper districts and also had higher serum urea levels, less frequent Delta variant infection, higher SAPS II and SOFA scores and day-28 mortality rates. WHO World Health Organization, SOFA Sequential Organ Failure Assessment, SAPS II Simplified Acute Physiology Score II, MV mechanical ventilation

The original article has been corrected.
