# Supplementary material for: COVID-19 associated pulmonary aspergillosis in critically-ill patients: a prospective multicenter study in the era of Delta and Omicron variants
Source: Ann Intensive Care. 2024 Apr 24;14:65. doi: 10.1186/s13613-024-01296-0 (PMC11043290; doi:10.1186/s13613-024-01296-0)
Supplement: Supplementary file 1 — Additional file 1: Figure S1. Diagnosis criteria of CAPA (proven/probable and possible), relied on ECMM/ISHAM consensus criteria. BAL, bronchoalveolar lavage; CAPA, COVID-19-associated pulmonary aspergillosis; PCR, polymerase chain reaction. [file 13613_2024_1296_MOESM1_ESM.pptx]

## Slide 1
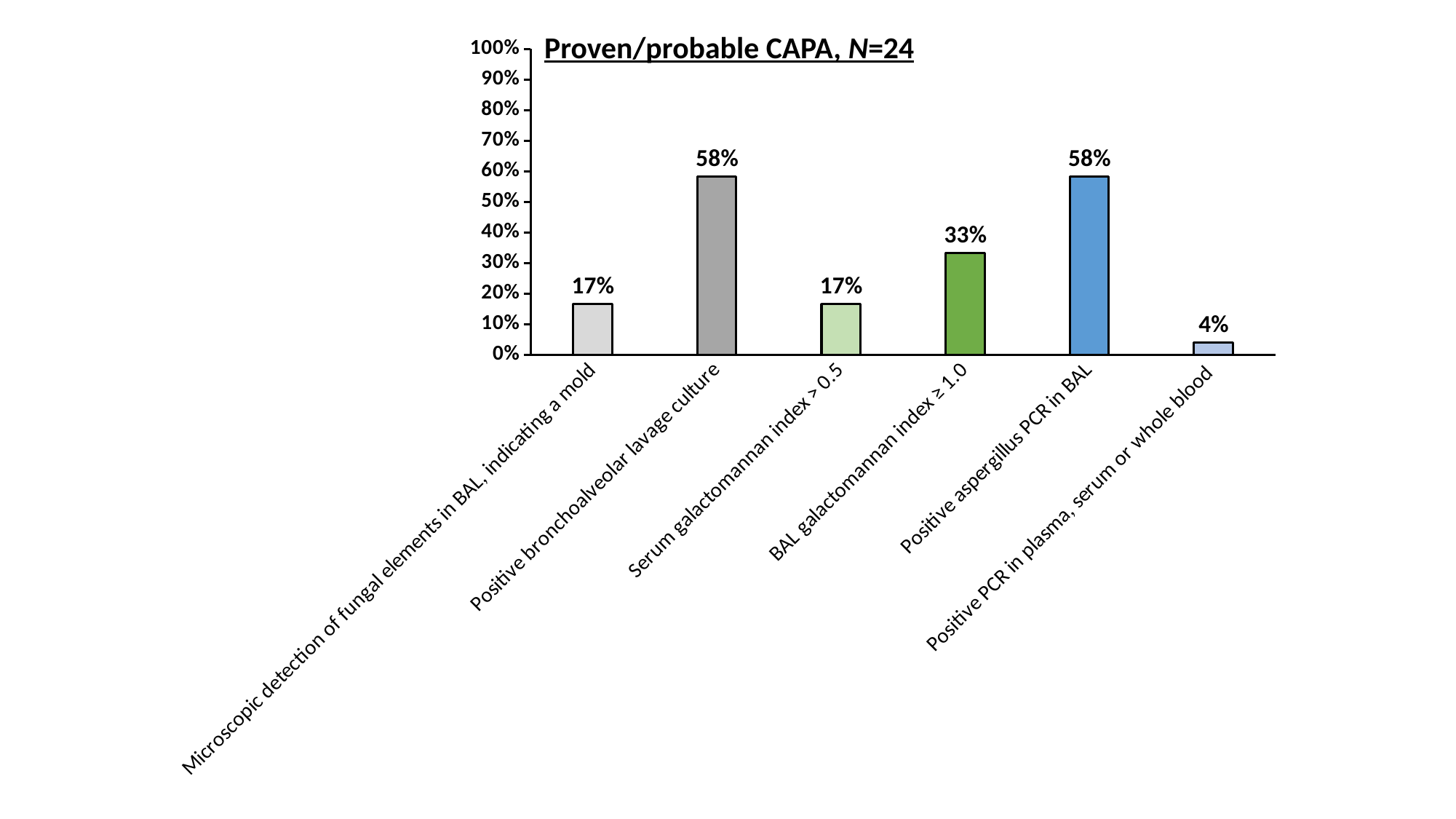

### Chart
| Category | Colonne1 |
|---|---|
| Microscopic detection of fungal elements in BAL, indicating a mold | 0.166666667 |
| Positive bronchoalveolar lavage culture | 0.583333333 |
| Serum galactomannan index > 0.5 | 0.166666667 |
| BAL galactomannan index ≥ 1.0 | 0.333333333 |
| Positive aspergillus PCR in BAL | 0.583333333 |
| Positive PCR in plasma, serum or whole blood | 0.041666667 |Proven/probable CAPA, N=24

## Slide 2
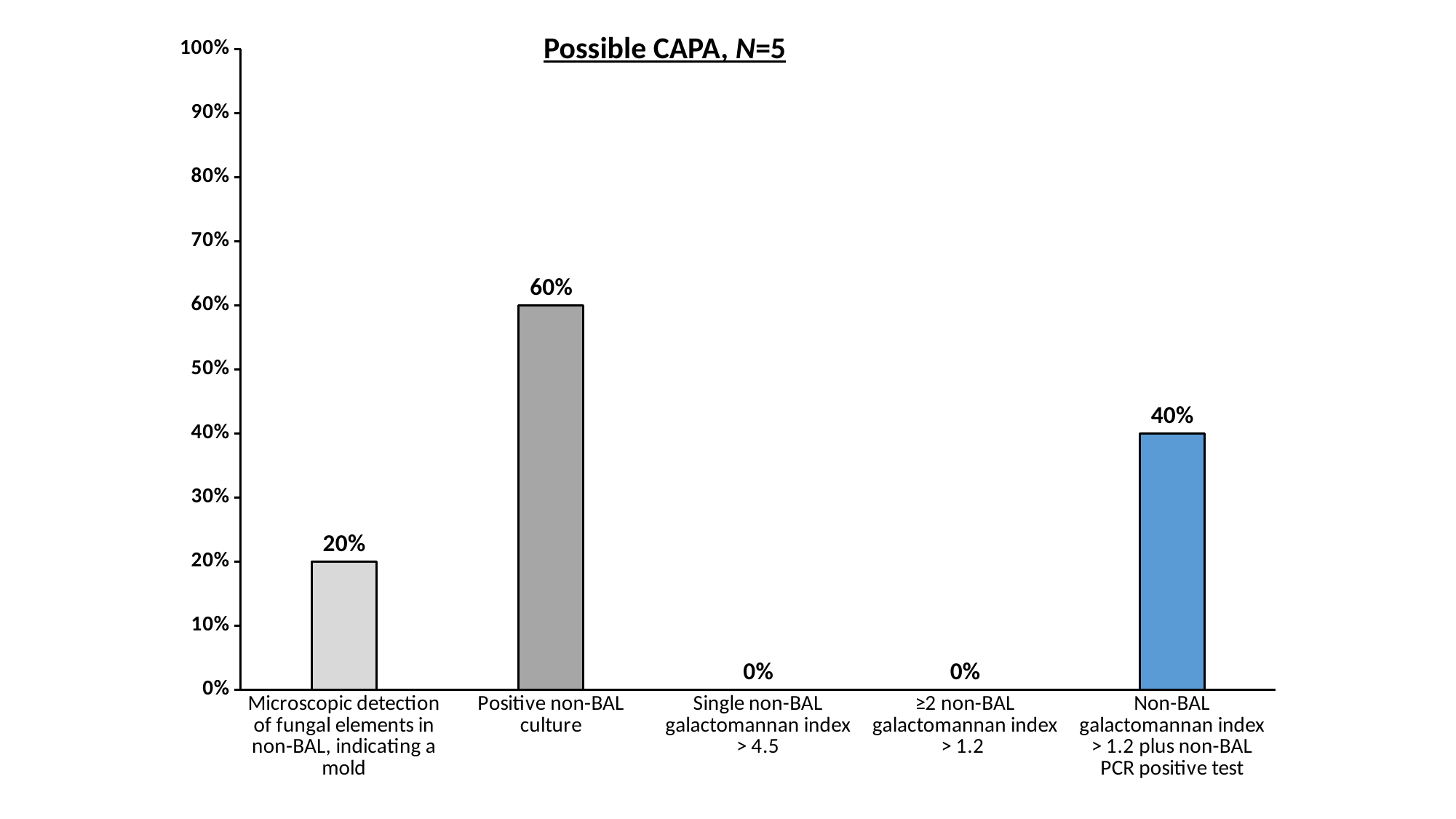

### Chart
| Category | Colonne1 |
|---|---|
| Microscopic detection of fungal elements in non-BAL, indicating a mold | 0.2 |
| Positive non-BAL culture | 0.6 |
| Single non-BAL galactomannan index > 4.5 | 0.0 |
| ≥2 non-BAL galactomannan index > 1.2 | 0.0 |
| Non-BAL galactomannan index > 1.2 plus non-BAL PCR positive test | 0.4 |Possible CAPA, N=5
