# Supplementary material for: COVID-19 associated pulmonary aspergillosis in critically-ill patients: a prospective multicenter study in the era of Delta and Omicron variants
Source: Ann Intensive Care. 2024 Apr 24;14:65. doi: 10.1186/s13613-024-01296-0 (PMC11043290; doi:10.1186/s13613-024-01296-0)
Supplement: Supplementary file 2 — Additional file 2: Table S1. Criteria used for the classification of patients according to the ECMM/ISHAM consensus criteria. [file 13613_2024_1296_MOESM2_ESM.docx]

| **Table S1.** Criteria used for the classification of patients according to the ECMM/ISHAM consensus criteria. | | |
| --- | --- | --- |
|  | CAPA proven/probable | CAPA possible |
| Clinical | At least one of the following:   - Refractory fever - Pleural rub - Chest pain - Hemoptysis | At least one of the following:   - Refractory fever - Pleural rub - Chest pain - Hemoptysis |
| Imaging | All ARDS patients had at least one chest CT as part of their routine follow up that showed pulmonary infiltrate, cavitating infiltrate, or other CT patterns that can be due to either the underlying COVID-19 condition or a secondary infection. | |
| Mycology | At least one of the following:   - Microscopic detection of fungal elements in bronchoalveolar lavage, indicating a mold - Positive bronchoalveolar lavage culture - Serum galactomannan index >0.5 or serum LFA index >0.5‡ - Bronchoalveolar lavage galactomannan index ≥1.0 or bronchoalveolar lavage LFA index ≥1.0 - Two or more positive Aspergillus PCR tests in plasma, serum, or whole blood - A single positive Aspergillus PCR in bronchoalveolar lavage fluid (<36 cycles) - A single positive Aspergillus PCR in plasma, serum, or whole blood, and a single positive in bronchoalveolar lavage fluid (any threshold cycle permitted) | At least one of the following:   - Microscopic detection of fungal elements in non-bronchoscopic lavage indicating a mold - Positive non-bronchoscopic lavage culture - Single non-bronchoscopic lavage galactomannan index >4.5 - Non-bronchoscopic lavage galactomannan index >1.2 twice or more - • Non-bronchoscopic lavage galactomannan index >1.2 plus another positive nonbronchoscopic lavage mycology test (nonbronchoscopic lavage PCR or LFA) |
