# Supplementary material for: COVID-19 associated pulmonary aspergillosis in critically-ill patients: a prospective multicenter study in the era of Delta and Omicron variants
Source: Ann Intensive Care. 2024 Apr 24;14:65. doi: 10.1186/s13613-024-01296-0 (PMC11043290; doi:10.1186/s13613-024-01296-0)
Supplement: Supplementary file 3 — Additional file 3: Table S2. Predictors of CAPA occurrence by univariable and multivariable logistic regression models in critically ill patients with COVID-19: results on raw data (n = 566). [file 13613_2024_1296_MOESM3_ESM.docx]

| **Table S2. Predictors of CAPA occurrence by univariable and multivariable logistic regression models in critically ill patients with COVID-19: results on raw data (n=566).** | | | | | | | | | |
| --- | --- | --- | --- | --- | --- | --- | --- | --- | --- |
|  | **Univariable Analysis** | | |  | | **Multivariable Analysis** | | | |
| **Factor** | **OR** | **95%CI** | **p-value** | |  | | **aOR** | **95%CI** | **p-value** |
| Blood leukocytes, G/L | 1.02 | (1.00;1.05) | **0.019** | |  | |  |  |  |
| SAPS II score | 1.02 | (1.00;1.05) | **0.043** | |  | | **1.03** | **(1;1.06)** | **0.03** |
| Age, years | 1.00 | (0.98;1.03) | 0.74 | |  | |  |  |  |
| Gender, females | 0.74 | (0.32;1.70) | 0.473 | |  | |  |  |  |
| SOFA score | 1.12 | (1.00;1.25) | **0.041** | |  | |  |  |  |
| Serum urea level, mM | 1.01 | (0.99;1.03) | 0.384 | |  | |  |  |  |
| First symptoms - ICU admission, days | 1.01 | (1.00;1.06) | **0.043** | |  | | **1.03** | **(1-1.06)** | **0.024** |
| Invasive mechanical ventilation | 2.02 | (0.93;4.38) | 0.077 | |  | |  |  |  |
| Immunosuppression | 2.28 | (1.06;4.90) | **0.035** | |  | |  |  |  |
| SARS-CoV-2 variant |  |  |  | |  | |  |  |  |
| Omicron (ref) | 1 (ref) |  |  | |  | | 1 (ref) |  |  |
| Delta | 2.01 | (0.85;4.75) | 0.112 | |  | | 2.35 | (0.97-5.73) | 0.059 |
| aOR (CI 95%): adjusted Odds Ratio (95% confidence interval).  *CAPA,* COVID-19-associated pulmonary aspergillosis, *ICU* intensive care unit, *SAPS*, simplified acute physiology score; *SOFA* Sequential Organ Failure Assessment.  p-values come from multivariable logistic regression models | | | | | | | | | |
